# Upper Respiratory Symptoms as Long COVID: Insight from a Multicenter Cohort Study

**DOI:** 10.1002/oto2.120

**Published:** 2024-03-03

**Authors:** Masahiko Okada, Noriyuki Ishida, Sho Kanzaki, Ichiro Kawada, Kengo Nagashima, Hideki Terai, Gaku Hiruma, Ho Namkoong, Takanori Asakura, Katsunori Masaki, Keiko Ohgino, Jun Miyata, Shotaro Chubachi, Nobuhiro Kodama, Shunsuke Maeda, Satoshi Sakamoto, Masaki Okamoto, Yoji Nagasaki, Akira Umeda, Kazuya Miyagawa, Hisato Shimada, Kazuhiro Minami, Rie Hagiwara, Makoto Ishii, Yasunori Sato, Koichi Fukunaga

**Affiliations:** ^1^ Department of Medicine, Division of Pulmonary Medicine Keio University School of Medicine Tokyo Japan; ^2^ Biostatistics Unit, Clinical and Translational Research Center Keio University Hospital Tokyo Japan; ^3^ Department of Otorhinolaryngology–Head and Neck Surgery Keio University Tokyo Japan; ^4^ Laboratory of Auditory Disorders, National Institute of Sensory Organs National Hospital Organization Tokyo Medical Center Tokyo Japan; ^5^ Health Center Keio University Yokohama Japan; ^6^ Keio Cancer Center Keio University School of Medicine Tokyo Japan; ^7^ Department of Infectious Diseases Keio University School of Medicine Tokyo Japan; ^8^ Department of Respiratory Medicine Kitasato University Kitasato Institute Hospital Tokyo Japan; ^9^ Department of General Internal Medicine Fukuoka Tokushukai Hospital Fukuoka Japan; ^10^ Department of Respirology National Hospital Organization Kyushu Medical Center Fukuoka Japan; ^11^ Department of Infectious Disease and Clinical Research Center National Hospital Organization Kyushu Medical Center Fukuoka Japan; ^12^ Department of General Medicine, School of Medicine, IUHW Shioya Hospital International University of Health and Welfare (IUHW) Tochigi Japan; ^13^ Department of Pharmacology International University of Health and Welfare Tochigi Japan; ^14^ Department of Respiratory Medicine International University of Health and Welfare Shioya Hospital Tochigi Japan; ^15^ Department of Internal Medicine Saitama Medical Center Saitama Japan; ^16^ Department of Respiratory Medicine Nagoya University Graduate School of Medicine Nagoya Japan; ^17^ Department of Preventive Medicine and Public Health Keio University School of Medicine Tokyo Japan

**Keywords:** COVID‐19, long COVID, olfactory disorder, taste disorder, upper respiratory symptoms

## Abstract

**Objective:**

This study aimed to investigate the clinical features of long COVID cases presenting with upper respiratory symptoms, a topic not yet fully elucidated.

**Study Design:**

Prospective cohort study.

**Setting:**

A multicenter study involving 26 medical facilities in Japan.

**Methods:**

Inclusion criteria were patients aged ≥18 years old with a confirmed COVID‐19 diagnosis via severe acute respiratory syndrome coronavirus 2 polymerase chain reaction or antigen testing, who were hospitalized at the participating medical facilities. Analyzing clinical information and patient‐reported outcomes from 1009 patients were analyzed. The outcome measured the degree of initial symptoms for taste or olfactory disorders and assessed the likelihood of these symptoms persisting as long COVID, as well as the impact on quality of life if the upper respiratory symptoms persisted as long COVID.

**Results:**

Patients with high albumin, low C‐reactive protein, and low lactate dehydrogenase in laboratory tests tended to experience taste or olfactory disorders as part of long COVID. Those with severe initial symptoms had a higher risk of experiencing residual symptoms at 3 months, with an odds ratio of 2.933 (95% confidence interval [CI], 1.282‐6.526) for taste disorders and 3.534 (95% CI, 1.382‐9.009) for olfactory disorders. Presence of upper respiratory symptoms consistently resulted in lower quality of life scores.

**Conclusion:**

The findings from this cohort study suggest that severe taste or olfactory disorders as early COVID‐19 symptoms correlate with an increased likelihood of persistent symptoms in those disorders as long COVID.

COVID‐19 was first reported in China in December 2019,^1^ and rapidly spread worldwide, including in Japan. Despite the reduced mortality rate owing to improved treatments and vaccines, the number of affected patients continues to rise. As the number of patients increases, so does the number of individuals experiencing long‐term effects known as long COVID.^2^

Long COVID comprises fatigue, upper and lower respiratory symptoms, gastrointestinal and neurological symptoms.^3^, ^4^ Taste and olfactory disorders are commonly associated with an initial COVID‐19 diagnosis during the acute phase^5^, ^6^, ^7^, ^8^; however, they can persist as part of long COVID.^9^ At the onset of COVID‐19, upper respiratory symptoms, including sore throat, taste disorders, and olfactory disorders, have been identified as predictors of mild disease severity.^10^, ^11^ These symptoms are also frequently reported among individuals experiencing long COVID.^3^, ^4^, ^12^, ^13^, ^14^, ^15^ Several risk factors, such as younger age, milder COVID‐19 cases, and female sex, have been reported for persisting taste and olfactory disorders^16^, ^17^, ^18^, ^19^, ^20^, ^21^, ^22^; however, none have been definitively established.

Additionally, there is a lack of comprehensive assessments, including quality of life (QOL) indicators, regarding the influence of persistent upper respiratory symptoms that are typically not considered severe, in daily life. Given the limited research on cross‐sectional studies concerning upper respiratory symptoms in Japan and other countries, this study aims to investigate the development and characteristics of these upper respiratory symptoms in long COVID.

## Methods

### Study Design and Participants

This study was part of a comprehensive research project on long COVID designed as a multicenter prospective cohort study. The detailed protocol^23^ and initial analysis results^24^ for this project have already been published. For the present analysis, a multicenter cohort study was conducted from November 2020 to March 2022 involving patients with COVID‐19 registered at 26 medical facilities in Japan.

The study included patients aged ≥18 years who agreed to participate, had a confirmed diagnosis of COVID‐19 determined by severe acute respiratory syndrome coronavirus 2 (SARS‐CoV‐2) polymerase chain reaction or antigen testing, and were hospitalized at the participating medical facilities. Patients with language impairments or cognitive or psychiatric disorders that hindered their understanding of informed consent or ability to read and respond to questionnaires were excluded.

### Procedures

The participating medical facilities provided patients with a study information sheet and obtained their consent to participate. Subsequently, patient information was registered in the case information registration system, and patients completed the patient‐reported outcomes using paper forms or a smartphone application. Upon enrollment, the investigator collected clinical information, including age, sex, height, weight, vital signs at hospital admission, pre‐existing medical conditions, and blood test results. The participants were assigned unique identification numbers generated by the participating facilities, ensuring responses did not include any personal identifiable information. This study was approved by the Keio University School of Medicine Ethics Committee (approval No. 20200243) and the ethical committees of each participating institution. Additionally, it was registered in the clinical trial registration system (UMIN ID:000042299).

### Original Questionnaire

This study used an original questionnaire^24^ comprising comprehensive surveys regarding symptoms that appeared at the time of COVID‐19 diagnosis, duration, time of onset, severity, and sequelae. The symptoms included sore throat, taste, and olfactory disorders, defined as upper respiratory symptoms. We also assessed symptom improvement, recurrences post‐COVID‐19 onset, and new symptoms that appeared after discharge. Symptoms at admission were retrospectively investigated using a questionnaire at 3 months, repeated at 3, 6, and 12 months. Patients who experienced taste or olfactory disorders during hospitalization were requested to indicate the severity of the disorder at its worst, providing a taste or smell score on a questionnaire at 3 months, ranging from 0 (complete loss of taste or smell) to 10 (normal). Patients with olfactory disorders followed the COVID‐19 anosmia reporting tool.^25^ QOL were conducted at 3, 6, and 12 months, tabulated using the EuroQol 5 Dimensions 5‐Level, (EQ‐5D‐5L),^26^ Short Form‐8 (SF‐8).^27^

### Variable Definitions

We defined oxygen requirement during hospitalization as follows: patients with positive, severe, moderate II and negative, mild, moderate I.^28^ We classified age into 3 categories: young (18‐40 years), middle‐aged (41‐64 years), and older adults (>65 years).^24^ We classified body mass index (BMI) into 4 categories: underweight (<18.5), normal range (18.5‐24.99), preobese (25‐29.99), and obese (30≤).^29^

### Statistical Analyses

The patients' baseline characteristics are presented as medians and interquartile ranges for continuous variables and as numbers and percentages for categorical variables. To examine the association between the presence of long COVID and comorbidities, longitudinal analyses were performed using a generalized linear mixed model with a logit link function, assuming an unstructured covariance matrix for each time point. In cases of non‐convergence, the covariance structure was progressively simplified in the order of Toeplitz, heterogeneous compound symmetry, first‐order autoregressive, compound symmetry, and variance components until convergence was achieved. We compared the EQ‐5D‐5L and SF‐8 scores between the groups using Mann‐Whitney *U* test. A 2‐sided significance level of 5% was used for all statistical hypothesis tests. All analyses were performed using the SAS statistical software (v.9.4), GraphPad Prism (v 9.51 MAC), and R (R Core Team, 2022) R: Language and environment for statistical computing. R Foundation for Statistical Computing). The data were visualized using GraphPad Prism and R.

## Results

### Comparison of Baseline Characteristics of Patients With or Without Upper Respiratory Symptoms

Out of the 1200 patients registered in this study, 134 were excluded from the background analysis due to the incomplete medical records and 57 were excluded as they were unresponsive to all 3 items at time of admission (sore throat, taste disorder, and olfactory disorder). Consequently, 1009 were included in the analysis for this project (Supplemental Figure S1, available online).

Among the patients who experienced taste disorders at least once (n = 355), 314 provided their taste disorder scores. It is uncommon for smell or taste problems to develop at a later stage if no issues were observed during the initial phase of infection.^13^, ^20^, ^30^, ^31^ Therefore, we excluded cases with late‐onset olfactory and taste disorders from the analysis. After excluding patients who did not report taste symptoms during hospitalization, the taste disorder scores of 292 individuals were analyzed. Similarly, among patients who manifested olfactory disorders at least once (n = 304), 282 provided olfactory scores. After excluding patients without olfactory symptoms during hospitalization, the olfactory scores of 262 individuals were analyzed (Supplemental Figure S2, available online). Baseline characteristics of this study were summarized in Table 1 and Supplemental Table S1, available online.

**Table 1:** Baseline Characteristics of all Patients (n = 1009)

Variables	All (n = 1009)	Sore throat (n = 35)	Taste disorder (n = 63)	Olfactory disorder (n = 79)
Severity, N (%)				
Oxygen not required	659 (65.3)	21 (60.0)	49 (77.8)	65 (82.3)
Oxygen required	326 (32.3)	14 (40.0)	12 (19.0)	11 (13.9)
Missed	24 (2.4)	0 (0.0)	2 (3.2)	3 (3.8)
Age, mean, (SD)	56.77 (16.64)	56.74 (15.21)	50.89 (19.12)	50.70 (16.86)
Age group, N (%)				
Young	192 (19.0)	8 (22.8)	24 (38.1)	25 (31.6)
Middle‐aged	461 (45.7)	17 (48.6)	23 (36.5)	37 (46.8)
Older adults	354 (35.1)	10 (28.6)	16 (25.4)	17 (21.5)
Missed	2 (0.2)	0	0	0
BMI, N (%)				
Underweight	49 (4.9)	2 (5.7)	4 (6.3)	8 (10.1)
Normal	538 (53.3)	20 (57.1)	40 (63.5)	48 (60.8)
Preobese	283 (28.0)	7 (20.0)	10 (15.9)	14 (17.7)
Obese	80 (7.9)	4 (11.4)	5 (7.9)	3 (3.8)
Missed	59 (5.8)	2 (5.7)	4 (6.3)	6 (7.6)
Sex, N (%)				
Male	639 (63.3)	23 (65.7)	30 (47.6)	31 (39.2)
Female	370 (36.7)	12 (34.3)	33 (52.4)	48 (60.8)

Abbreviations: BMI, body mass index; SD, standard deviation.

### Trend in the Percentage of Patients Affected with Each Symptom

**Figure 1:**
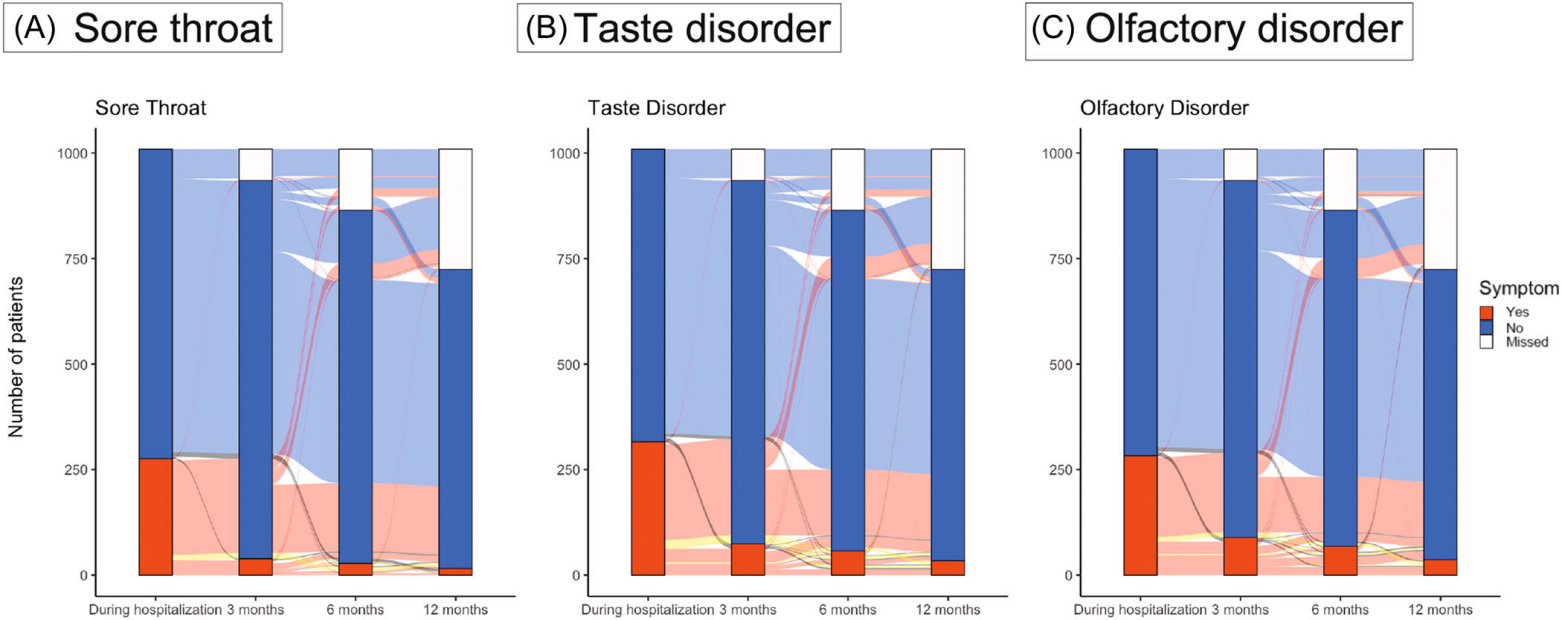
Trends of patients with upper respiratory symptoms. The blue curve shows asymptomatic individuals, the red curve shows symptom development during hospitalization, the yellow curve shows “relapse cases,” and the black curve depicts “late‐onset cases.”

Figure 1A‐C illustrates the trends in symptom percentages among the patients. We classified cases as “relapse cases” when patients initially experienced symptoms during hospitalization, showed temporary improvement, and later experienced symptom recurrence. Additionally, we classified cases as “late‐onset cases” when patients did not exhibit symptoms during their hospital stay but developed symptoms after being discharged. Based on reports suggesting the possibility of a relapse of taste and olfactory disorders associated with COVID‐19, we included relapse cases in our analysis.^32^ Late‐onset cases were not counted as long COVID in this analysis as detailed in previous section. The number of patients with each symptom decreased over time.

### Overlap of Patients With Each Symptom

**Figure 2:**
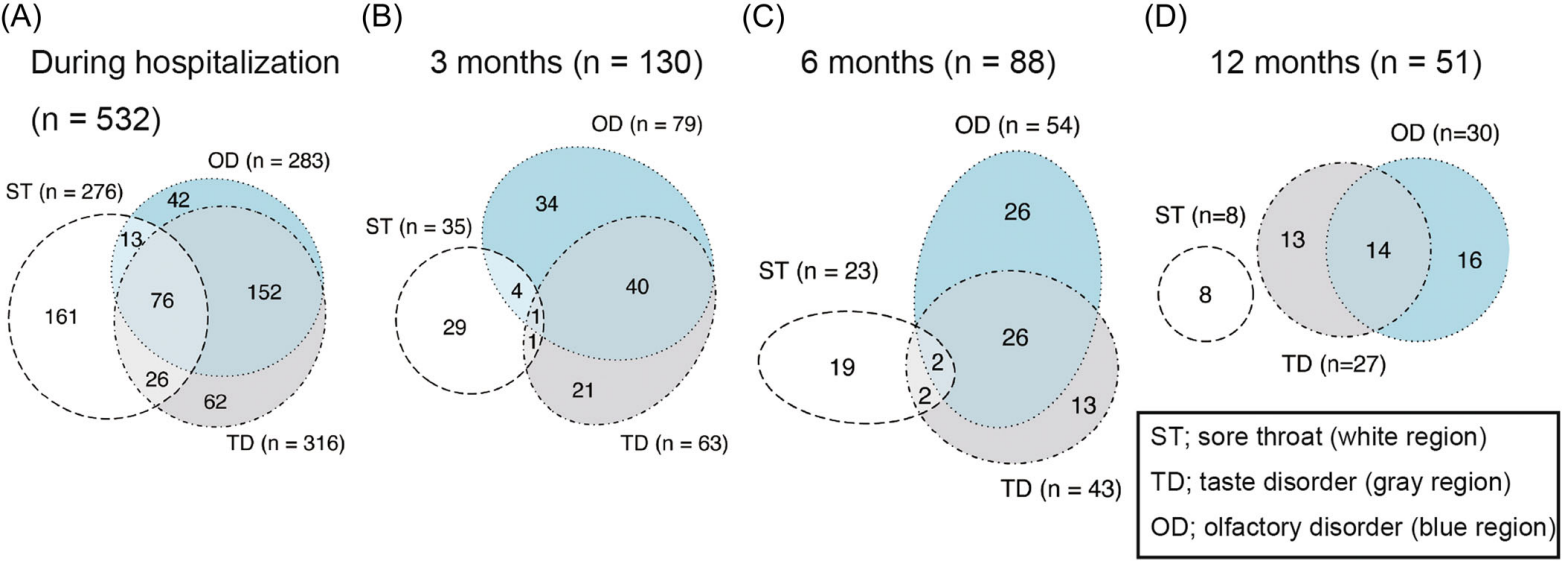
Distribution of patients with overlapping symptoms during hospitalization (A), and at 3 (B), 6 (C), and 12 months (D). OD, olfactory disorder; ST, sore throat; TD, taste disorder.

The results of symptoms co‐occurrence are presented in Figure 2A‐D and Supplemental Table S2, available online. Similar to Figure 1A‐C, the 3 symptoms improved over time. Initially, 267 (50.2%) patients experienced 2 or 3 upper respiratory symptoms during hospitalization. However, as time passed, the number of patients reporting sore throat decreased. Simultaneously, the occurrence of sore throat and the other 2 symptoms, also declined: 115 patients (21.6%) during hospitalization, 6 (4.6%) at 3 months, 4 (7.4%) at 6 months, and 0 (0%) at 12 months. Among patients with upper respiratory symptoms during hospitalization, 276 (51.9%) had sore throats, while only 8 (15.7%) had sore throats at 12 months. In contrast, the proportion of patients with taste and olfactory disorders slowly decreased over time; 228 patients (42.9%) had taste and olfactory disorders during hospitalization and 14 (27.5%) had both disorders at 12 months.

### Risk Factors of Each Symptom

**Figure 3:**
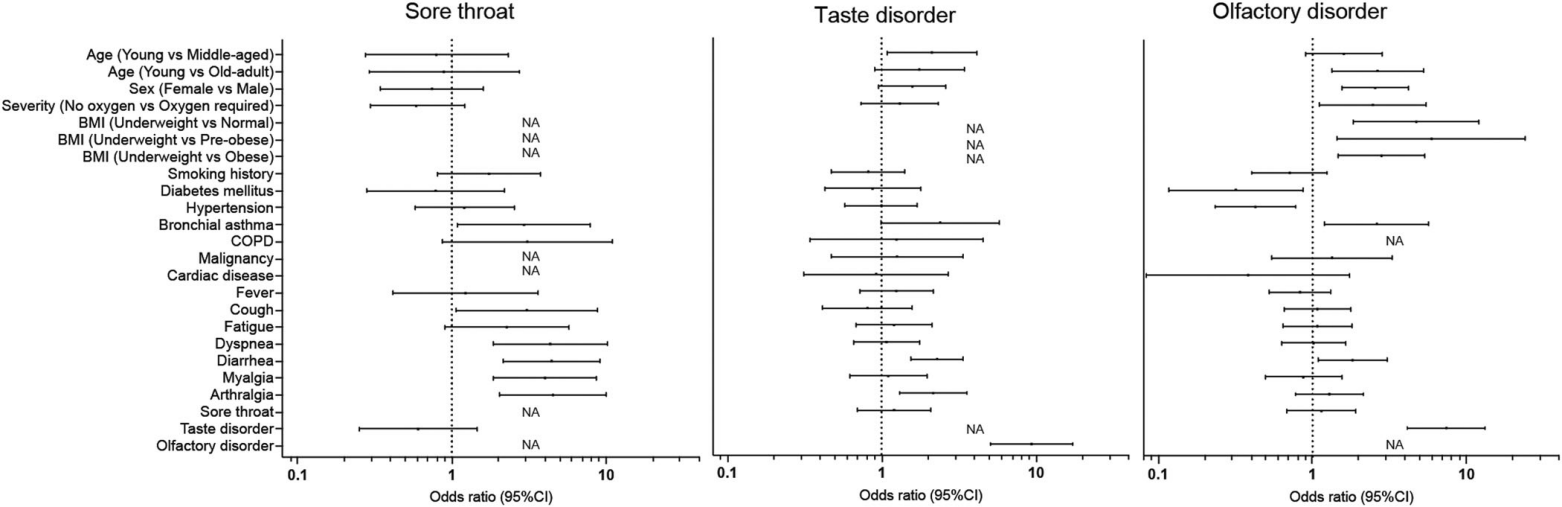
Risk factors for having upper respiratory symptoms at any point during 3, 6, and 12 months. BMI, body mass index; CI, confidence interval; NA, not available.

To identify the risk factors for each symptom, we performed univariate analysis with variables selected based on previous studies. These included age, sex, BMI, coexisting disease, severity of COVID‐19, COVID‐19‐related symptoms during hospitalization, and blood test findings upon admission.^10^, ^16^, ^17^, ^19^, ^20^, ^21^, ^22^ Additionally, we studied the link between endotracheal intubation and lingering sore throat, as well as the association between taste or olfactory disorders and their respective treatments. The results are presented in Figure 3, and Supplemental Tables S3‐7, available online.

### Sore Throat

The study findings indicated that the patients who experienced diarrhea, muscle pain, and joint pain during hospitalization were more likely to develop long‐term sore throat, with odds ratios of 4.431 (95% confidence interval [CI]: 2.152‐9.122), 4.005 (95% CI: 1.860‐8.625), and 4.518 (95% CI: 2.044‐9.987), respectively. Laboratory tests revealed high lactate dehydrogenase levels as risk factor for sore throat, with an odds ratio of 1.004 (95% CI: 1.001‐1.006). At 3 months, patients with sore throats tended to experience a more severe disease at the onset of COVID‐19 than the overall population. Patients who underwent endotracheal intubation tended to experience residual sore throat at 3 months, but the difference was not significant at 6 or 12 months, with odds ratios of 4.674 (95% CI: 1.828‐11.952), 2.379 (95% CI: 0.370‐8.626), and 6.210 (95% CI: 1.209‐31.884), respectively.

### Taste Disorder

Younger patients who experienced diarrhea, joint pain, and olfactory disorders during hospitalization were more likely to develop long‐term taste disorders, with odds ratios of 2.110 (young vs middle‐aged; 95% CI: 1.085‐4.115), 2.285 (95% CI: 1.553‐3.361), 2.153 (95% CI: 1.298‐3.570), and 9.326 (95% CI: 5.079‐17.125), respectively. High albumin, low hemoglobin A1c, low C‐reactive protein, and low lactate dehydrogenase levels in laboratory test were identified as risk factors for taste disorders as sequelae, with odds ratios of 1.615 (95% CI: 1.022‐2.553), 1.515 (95% CI: 1.038‐2.207), 1.076 (95% CI: 1.012‐1.144), and 1.004 (95% CI, 1.001‐1.007), respectively. No significant difference was observed in the treatment of taste disorder and its sequelae at 3, 6, and 12 months, with odds ratios of 1.607 (95% CI: 0.304‐8.484), 2.854 (95% CI: 0.506‐16.089), and 4.100 (95% CI: 0.714‐23.535).

### Olfactory Disorder

The risk factors for long COVID‐related olfactory disorder included younger age, female sex, lower BMI, milder COVID‐19 cases, and complications of diarrhea and taste disorder during hospitalization, with odds ratios of 2.645 (young vs older adults; 95% CI: 2.192‐5.208), 2.551 (95% CI: 1.557‐4.347), 2.463 (underweight vs normal; 95% CI: 1.106‐5.464), 2.808 (95% CI: 1.459‐5.4040), 1.818 (95% CI: 1.084‐3.049), and 7.412 (95% CI: 4.136‐13.281), respectively. Similar to the risk factors for taste disorders, high albumin, low hemoglobin A1c, low C‐reactive protein, and low lactate dehydrogenase levels were identified as risk factors for olfactory disorders as sequelae, with odds ratios of 2.598 (95% CI: 1.675‐4.028), 1.683 (95% CI: 1.116‐2.5440), 1.109 (95% CI: 1.041‐0.183), and 1.004 (95% CI: 1.002‐1.007), respectively. About characteristics at 3 months, patients with taste and smell disorders tended to have milder symptoms and were younger at the onset of COVID‐19 than overall population. At 6 months, patients treated for olfactory disorders showed a tendency toward residual symptoms; however, no significant differences were observed at 3 or 12 months, with odds ratios of 4.041 (95% CI: 1.125‐14.511), 2.708 (95% CI: 0.846‐8.669), and 3.423 (95% CI: 0.806‐14.530).

### Relationship Between the Taste/Smell Score and the Taste/Olfactory Disorder as Long COVID

**Figure 4:**
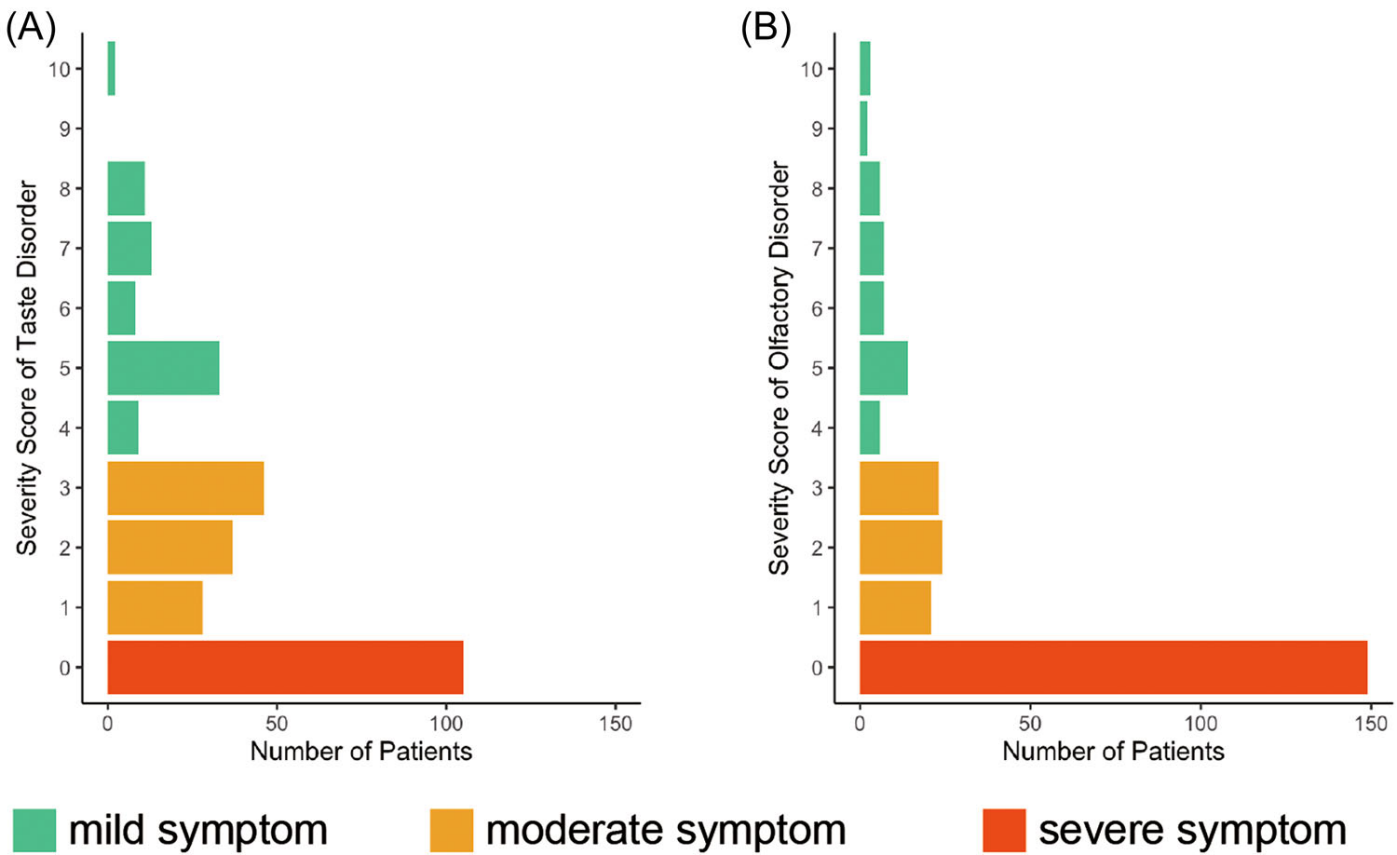
Number of patients with each score for severity questionnaire for taste disorders (A) or olfactory (B) disorders and relationship between each score and tendency for residual symptoms.

**Figure 5:**
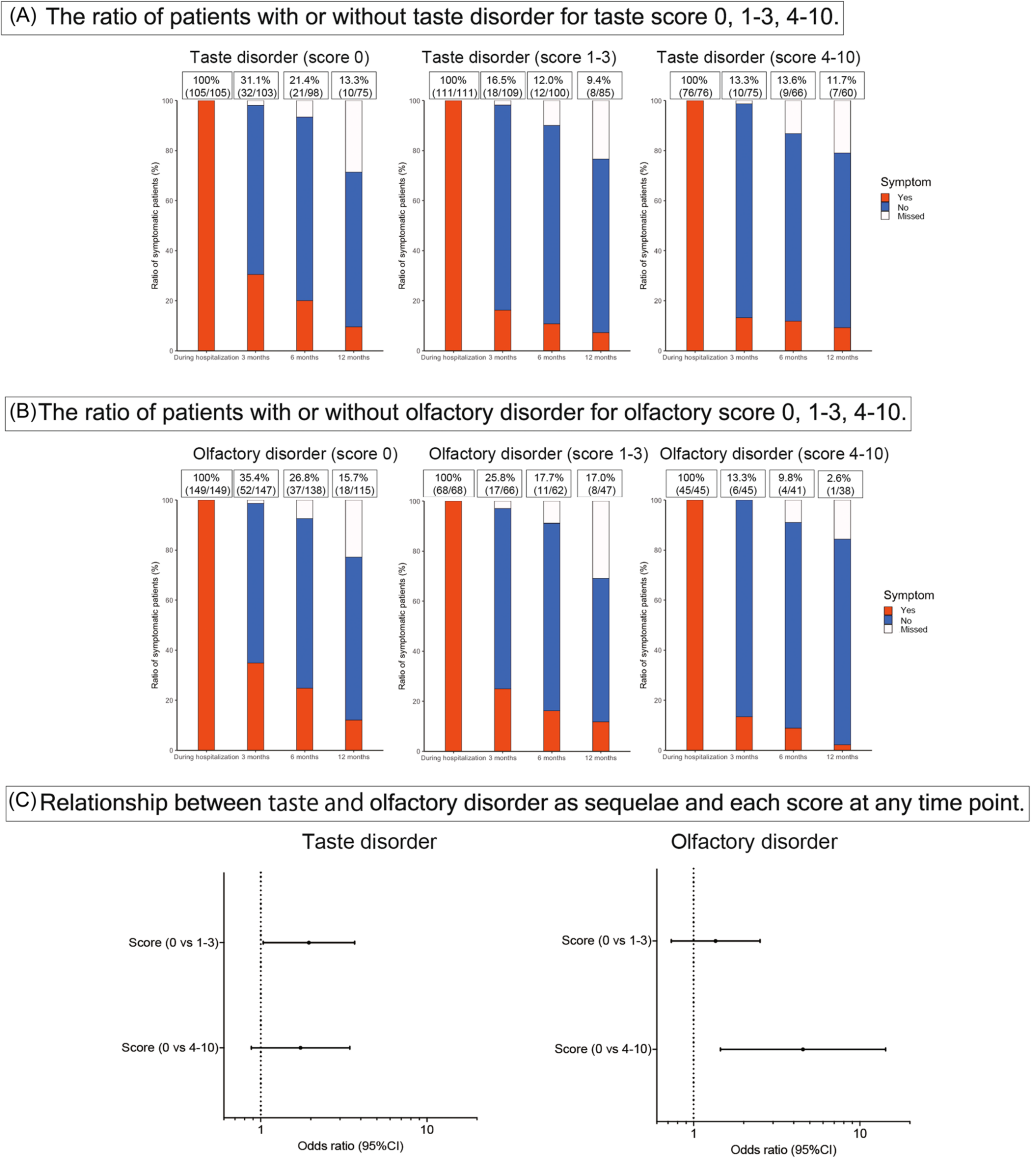
Relationship between scores and taste (A) or olfactory (B) disorders as sequelae, and odds ratios at any time points (C). CI, confidence interval.

In Figure 4, we display the numbers of patients who reported each score. To assess the severity of symptoms, we categorized the scores as follows: a score of 0 indicated a severe condition, scores 1, 2, and 3 represented moderate, and scores from 4 to 10 indicated mild. Most patients responded with a score of 0, indicating a complete loss of taste or smell at the onset of the disorder. Supplemental Table S8, available online and Figure 5A,B provide information on the correlation between scores and symptoms at 3, 6, and 12 months. For taste disorders, a score of 0 significantly correlated with persistent taste disorders at 3 months, with odds ratios of 2.262 (score 0 vs score 1‐3; 95% CI: 1.160‐4.405) and 2.932 (score 0 vs score 4‐10; 95% CI: 1.282‐6.535). Regarding olfactory disorders, severe cases (score 0) showed persistent symptoms at 3 and 6 months, compared with mild cases (score 4‐10), with odds ratios of 3.533 (95% CI: 1.381‐9.009) at 3 months, 3.546 (95% CI: 1.145‐10.989) at 6 months, and 4.545 (95% CI: 1.449‐14.285), respectively. Figure 5C illustrates the trends in symptoms for each score. Both taste and smell disorders, showed a tendency for a higher proportion of patients to experience symptoms at each time point when the symptoms were more severe at the onset.

### Relationship Between Presence of Symptoms and Their QOL

**Figure 6:**
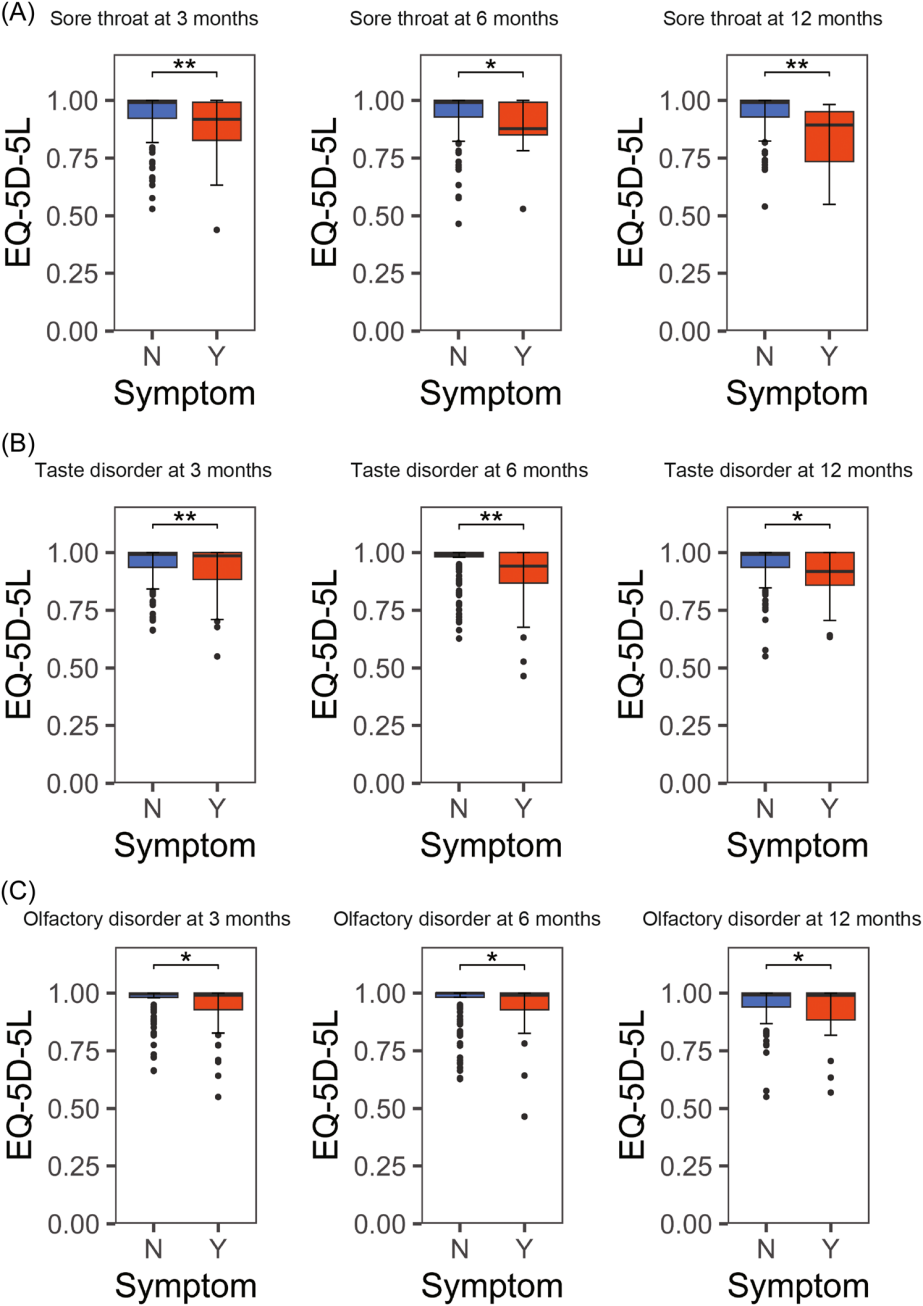
Relationship between EQ‐5D‐5L and sore throat (A), taste disorder (B), and olfactory disorder (C) as sequelae. *P < .05, **P < .01, and ***P < .001. EQ‐5D‐5L, EuroQol 5 dimensions 5‐level; N, no; Y, yes.

**Figure 7:**
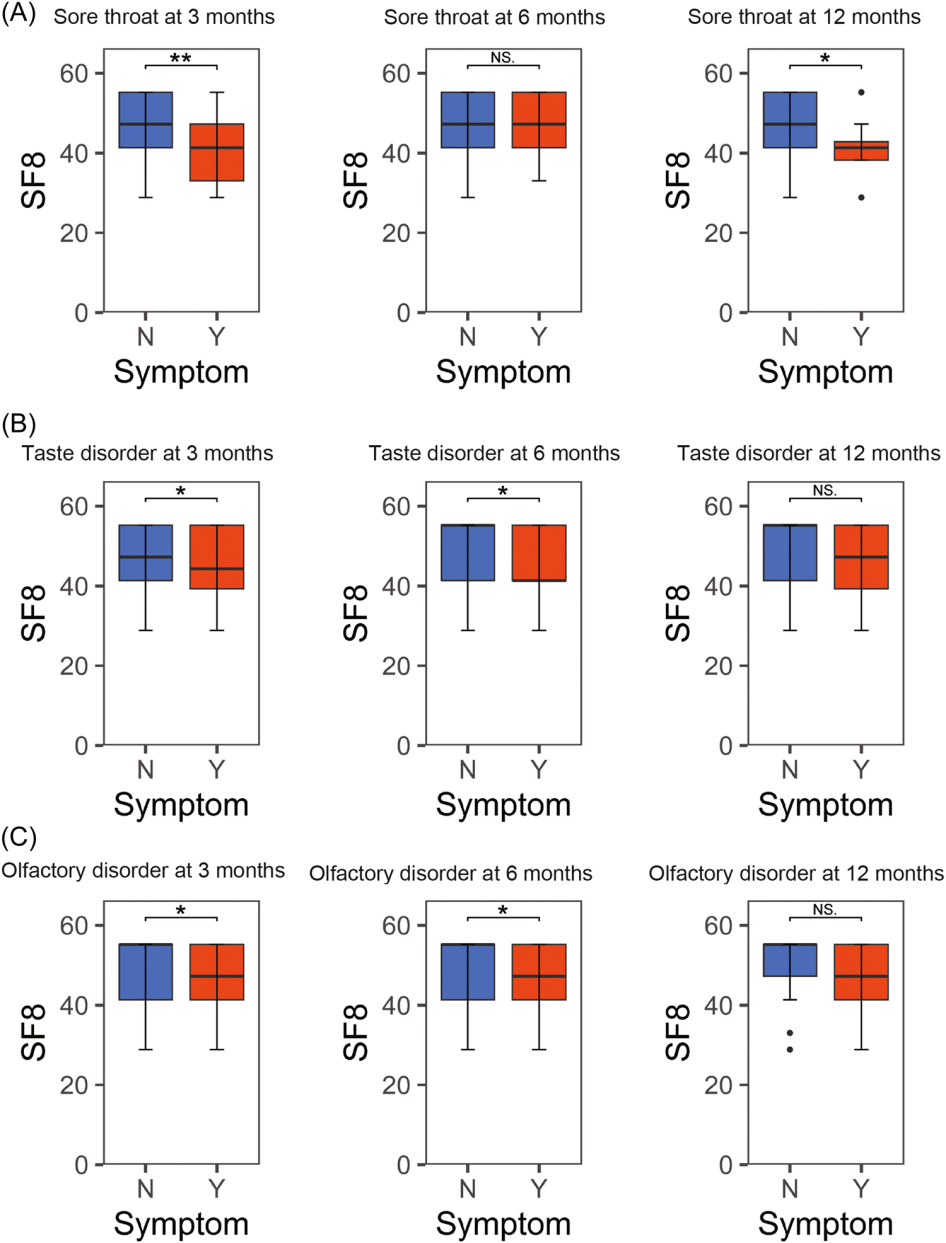
Relationship between SF‐8 and and sore throat (A), taste disorder (B), and olfactory disorder (C) as sequelae. *P < .05, **P < .01, and ***P < .001. SF‐8, short form‐8; N, no; Y, yes.

We compared the QOL using the EQ‐5D‐5L and SF‐8 scores to assess the impact of each symptom. The results are shown in Figures 6 and 7, and Supplemental Table S9, available online. The presence of upper respiratory symptoms consistently resulted in lower QOL scores.

## Discussion

This study utilized data from the largest long COVID symptom cohort study in Japan and performed an additional analysis focusing on upper respiratory symptoms. These symptoms are predictors of mild COVID‐19.^10^, ^11^ Three upper respiratory symptoms were often comorbid during hospitalization; however, over time, sore throat resolved and disappeared, while taste and olfactory disorders were comorbid at 12 months in some cases. Reports on olfactory disorders post‐COVID‐19 vary, indicating both high and low recovery rates.^33^, ^34^ Our data revealed that at least 10% of the patients experienced residual taste and olfactory disorders at 12 months.

We found that the risk factors differed among sore throat, taste, and olfactory disorders. Sore throat was associated with symptoms of viral illness, such as myalgia and arthralgia, during hospitalization. Younger age was a risk factor for taste disorders as long COVID, and younger age, milder COVID‐19, and lower body weight were associated with olfactory disorders. Our analysis revealed a tendency for patients who experienced diarrhea during hospitalization to develop 3 upper respiratory symptoms as long COVID. To our knowledge, no reports have discussed the relationship between upper respiratory symptoms and diarrhea as long COVID. However, existing studies emphasize the high specificity of diarrhea, taste, and smell disorders at onset for COVID‐19.^35^, ^36^, ^37^, ^38^ Another report suggests a correlation between taste and smell disorders and diarrhea.^39^ Although few risk factors for sore throat as long COVID have been studied in detail,^40^, ^41^, ^42^ myalgia, arthralgia, and diarrhea during hospitalization may be risk factors.

Additionally, we observed an association between tracheal intubation residual sore throat at 3 months, but not at 6 or 12 months. Since sore throat after tracheal intubation becomes rare after 10 days,^43^ the persistence of symptoms beyond 3 months would not be affected by the tracheal intubation itself. Our study also revealed that taste and smell disorders were more likely to persist as long COVID when C‐reactive protein was low or albumin was high at admission. This is consistent with previous findings,^44^ indicating that less severe cases are more likely to experience taste and smell disorders as sequelae.

In sequelae, as reported previously,^34^ we observed a mutual influence between taste and olfactory disorders, where the presence of one symptom during hospitalization increased the risk of others' persisting as long‐term effects. Regarding treatment, our findings revealed that treating taste and smell disorders had little impact on residual symptoms as aftereffects. Rather, treatment might result in residual symptoms as aftereffects. This could be attributed to the relatively small number of people treated for taste and smell disorders, as treatment for these disorders has not yet been firmly established.^45^, ^46^

Concerning taste and olfactory disorder scores, most patients responded with a score of 0, indicating that many experience complete loss of taste or smell after COVID‐19 infection. Reports indicate a higher likelihood of residual symptoms as long COVID with stronger symptoms at the onset of taste and olfactory disorders.^18^ In this study, we showed that the worse the score, the higher the likelihood of residual symptoms based on the degree of symptoms during hospitalization. For olfactory dysfunction, scores of 4 to 10 indicated a near‐complete disappearance of symptoms at 12 months. This suggested that if the symptoms of olfactory disorders were mild at onset, the probability of residual symptoms as long COVID was lower.

We demonstrated that the persistence of these upper respiratory symptoms as long COVID significantly reduced QOL. Many cases have developed symptoms of long COVID,^47^ which have been linked to decreased QOL; a crucial point in public health also included in our analysis.

One limitation of this study was the small sample size of symptomatic patients at each time point, primarily due to symptom improvement over time; this resulted in nonconvergence of the results when calculating the risk factors at different point. Consequently, only univariate analyses were feasible; multivariate analyses could not be effectively conducted. Second, regarding symptoms during hospitalization, the patient was retrospectively queried at 3 months, making it unclear whether these symptoms manifested at upon admission or later. Additionally, taste and olfactory disorders scoring was assessed only during hospitalization; the study did not track the changes in the symptoms over time at 3, 6, or 12 months. Consequently, it was not possible to evaluate the degree of improvement or track the progression of these symptoms beyond the hospitalization period. Furthermore, this study relied on self‐reported questionnaires to assess taste and olfactory disorders, and lacking objective measures. Although an objective evaluation method for olfactory or taste disorders has not been firmly established, it might be valuable to assess the correlation of the self‐reported scores utilized in this study with evaluation methods for olfactory and taste disorders that can be conducted in other clinical settings, as a complementary line of research in the future.

Despite these limitations, this study is an important contribution to the field, offering valuable insights into the trajectory of upper respiratory symptoms over 1 year post‐COVID‐19 hospitalization and the risks of persistent long COVID within a large cohort study.

## Conclusion

This study demonstrated that the severity of taste and olfactory disorders during hospitalization correlated with a higher likelihood of long COVID persistence. Additionally, the presence of upper respiratory symptoms, as long COVID, reduced the QOL of symptomatic patients. These findings suggest the potential for predicting prolonged taste and olfactory disorders as long COVID.

## Author Contributions

**Masahiko Okada**, contributed to the design, conduct, analysis, and drafting of the manuscript; **Noriyuki Ishida**, contributed to the design, conduct, analysis, and drafting of the manuscript; **Sho Kanzaki**, contributed to the design, conduct, analysis, and drafting of the manuscript; **Ichiro Kawada**, contributed to the design, conduct, analysis, and critical editing of the manuscript; **Kengo Nagashima**, contributed to the design, conduct, analysis, and critical editing of the manuscript; **Hideki Terai**, contributed to the design, conduct, analysis, and critical editing of the manuscript; **Gaku Hiruma**, contributed to the design and analysis of the manuscript; **Ho Namkoong**, contributed to the design and analysis of the manuscript; **Takanori Asakura**, contributed to the design and analysis of the manuscript; **Katsunori Masaki**, contributed to the design and analysis of the manuscript; **Keiko Ohgino**, contributed to the design and analysis of the manuscript, **Jun Miyata**, contributed to the design and analysis of the manuscript; **Shotaro Chubachi**, contributed to the design and analysis of the manuscript; **Nobuhiro Kodama**, contributed to the design and analysis of the manuscript; **Shunsuke Maeda**, contributed to the design and analysis of the manuscript; **Satoshi Sakamoto**, contributed to the design and analysis of the manuscript; **Masaki Okamoto**, contributed to the design and analysis of the manuscript; **Yoji Nagasaki**, contributed to the design and analysis of the manuscript; **Akira Umeda**, contributed to the design and analysis of the manuscript; **Kazuya Miyagawa**, contributed to the design and analysis of the manuscript; **Hisato Shimada**, contributed to the design and analysis of the manuscript; **Kazuhiro Minami**, contributed to the design and analysis of the manuscript, **Rie Hagiwara**, contributed to the design and analysis of the manuscript; **Makoto Ishii**, contributed to the critical editing of the manuscript; **Yasunori Sato**, contributed to the critical editing of the manuscript; **Koichi Fukunaga**, contributed to the critical editing of the manuscript.

## Disclosures

### Competing interests

None.

### Funding source

This research was funded by the Health Labor Science Special Research Project (20CA2054), and supported by AMED (JP20nk0101612, JP20fk0108415, JP20fk0108452, JP21fk0108553, JP21fk0108431, JP22fk0108510, JP21fk0108563, JP21fk0108573, JP22fk0108573, JP22fk0108513, JP22wm0325031), JST CREST (JPMJCR20H2), and JST PRESTO (JPMJPR21R7).

## Supporting information

Supporting information.

Supporting information.

Supporting information.

## Data Availability

The data that support the findings of this study are available on request from the corresponding author. The data are not publicly available due to privacy or ethical restrictions.
