# Supplementary figures and images for: Upper Respiratory Symptoms as Long COVID: Insight from a Multicenter Cohort Study
Source: OTO Open. 2024 Mar 3;8(1):e120. doi: 10.1002/oto2.120 (PMC10909391; doi:10.1002/oto2.120)

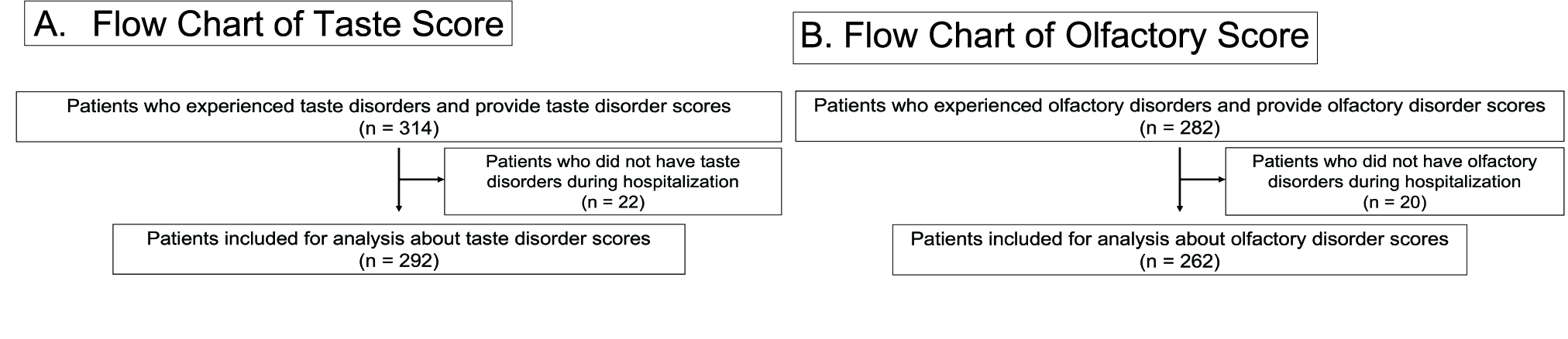

Supplement: Supplementary file 1 — Supporting information. [file OTO2-8-e120-s002.tif]

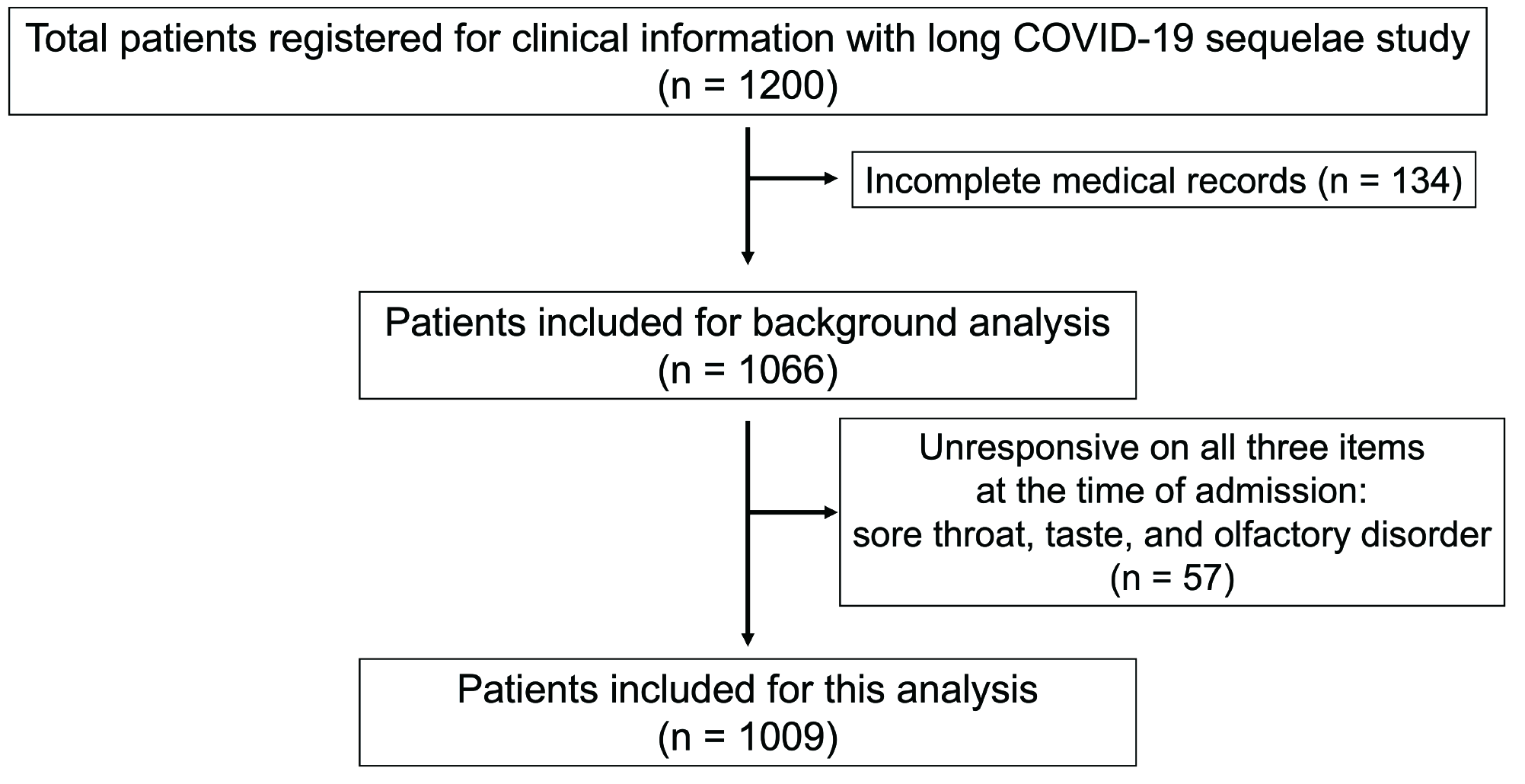

Supplement: Supplementary file 2 — Supporting information. [file OTO2-8-e120-s001.tif]
