# Supplementary material for: Upper Respiratory Symptoms as Long COVID: Insight from a Multicenter Cohort Study
Source: OTO Open. 2024 Mar 3;8(1):e120. doi: 10.1002/oto2.120 (PMC10909391; doi:10.1002/oto2.120)
Supplement: Supplementary file 3 — Supporting information. [file OTO2-8-e120-s003.docx]

**Online only materials**

**Supplementary Figure 1.** Flow chart of this study.

**Supplementary Figure 2.** Flow chart of symptom scores.

**Supplementary Table 1.** Base line characteristics including background diseases and laboratory data.

Cr, creatinine; ECMO, extracorporeal membrane oxygenation; NA, not available; SD, standard deviation; UA, uric acid; WBC, white blood cell

**Supplementary Table 2.** Proportion of overlap of the number of patients with each symptom.

**Supplementary Table 3.** Risk factors for upper airway symptoms at 3, 6, and 12 months.

BMI, body mass index; CI, confidence interval; Cr, creatinine; NA, not available; SD, standard deviation; UA, uric acid; WBC, white blood cell

**Supplementary Table 4.** Characteristics of patients who had each symptom at 3 months.

BMI , body mass index; NA, not available; SD, standard deviation; UA, uric acid

**Supplementary Table 5.** Relationship between sore throat and endotracheal intubation.

CI, confidence interval

**Supplementary Table 6.** Relationship between taste disorder and treatment for taste disorder.

CI, confidence interval

**Supplementary Table 7**. Relationship between olfactory disorder and treatment for olfactory disorder.

CI, confidence interval

**Supplementary Table 8.** Relationship between each score and symptom.

CI, confidence interval

**Supplementary Table 9.** Relationship between presence of symptom and QOL.

EQ-5D-5L, EuroQol 5 dimensions 5-level; IQR, interquartile range; SF-8, short form 8
